# Comparison of particle-exposure triggered pulmonary and systemic inflammation in mice fed with three different diets

**DOI:** 10.1186/1743-8977-8-30

**Published:** 2011-09-27

**Authors:** Alexander A Götz, Jan Rozman, Heiko G Rödel, Helmut Fuchs, Valérie Gailus-Durner, Martin Hrabě de Angelis, Martin Klingenspor, Tobias Stoeger

**Affiliations:** 1 Comprehensive Pneumology Center, Institute of Lung Biology and Disease, Helmholtz Zentrum München, German Research Center for Environmental Health, Ingolstädter Landstraße 1, Neuherberg/Munich, D-85764, Germany; 2 Molecular Nutritional Medicine, Else Kröner-Fresenius Center, TUM, Am Forum 5, Freising-Weihenstephan, D-85350, Germany; 3 Laboratory of Experimental and Comparative Ethology E.A. 4443 (LEEC), Université Paris 13, Sorbonne Paris Cité, F-93430 Villetaneuse, France; 4 Institute of Experimental Genetics, Helmholtz Zentrum München, German Research Center for Environmental Health, Ingolstädter Landstraße 1, 85764 Neuherberg/Munich, D-85764, Germany; 5 Chair for Experimental Genetics, TUM, Am Hochanger 8, Freising-Weihenstephan, D-85350, Germany

**Keywords:** diet, caloric, obesity, immune cell, bronchoalveolar lavage, inflammation, resolution, molecular

## Abstract

**Background:**

Obesity can be linked to disease risks such as diabetes and cardiovascular disorders, but recently, the adipose tissue (AT) macrophage also emerges as actively participating in inflammation and immune function, producing pro- and anti-inflammatory factors. Connections between the AT and chronic lung diseases, like emphysema and asthma and a protective role of adipocyte-derived proteins against acute lung injury were suggested.

In this study we addressed the question, whether a diet challenge increases the inflammatory response in the alveolar and the blood compartment in response to carbon nanoparticles (CNP), as a surrogate for ambient/urban particulate air pollutants.

**Methods:**

Mice were fed a high caloric carbohydrate-rich (CA) or a fat-rich (HF) diet for six weeks and were compared to mice kept on a purified low fat (LF) diet, respectively. Bronchoalveolar lavage (BAL) and blood samples were taken 24 h after intratracheal CNP instillation and checked for cellular and molecular markers of inflammation.

**Results and discussion:**

The high caloric diets resulted in distinct effects when compared with LF mice, respectively: CA resulted in increased body and fat mass without affecting blood cellular immunity. Conversely, HF activated the blood system, increasing lymphocyte and neutrophil counts, and resulted in slightly increased body fat content. In contrast to higher pro-inflammatory BAL Leptin in CA and HF mice, on a cellular level, both diets did not lead to an increased pro-inflammatory basal status in the alveolar compartment per se, nor did result in differences in the particle-triggered response. However both diets resulted in a disturbance of the alveolar capillary barrier as indicated by enhanced BAL protein and lactate-dehydrogenase concentrations. Systemically, reduced serum Adiponectin in HF mice might be related to the observed white blood cell increase.

**Conclusion:**

The increase in BAL pro-inflammatory factors in high caloric groups and reductions in serum concentrations of anti-inflammatory factors in HF mice, clearly show diet-specific effects, pointing towards augmented systemic inflammatory conditions. Our data suggest that extended feeding periods, leading to manifest obesity, are necessary to generate an increased susceptibility to particle-induced lung inflammation; although the diet-challenge already was efficient in driving pro-inflammatory systemic events.

## Introduction

Obesity and its common sequelae (e.g. type II diabetes and cardio vascular diseases) are a worldwide increasing health risk factor. The mechanisms resulting in excess storage of fat reserves depend on the over-consumption of dietary energy stimulated by high caloric and attractive food and/or reduced daily energy expenditure due to sedentary life-style [1-3]. The underlying physiological pathways and their genetic causes interacting with environmental factors still require substantial research efforts. Importantly, not only direct effects of increased adiposity e.g. on the sceleto-muscular system or insulin function need to be considered but also the sensitivity of obese patients to noxious substances resulting from air pollution or the adverse effects of very small airborne particles that are inhaled [1-3].

The adipose tissue (AT) is considered to actively participate in the regulation of physiologic and pathologic processes, like inflammation and immune function, and the obese state has been characterized to create systemic low-grade inflammation as indicated by increased inflammatory markers (reviewed in [4]). Among them are increased adipocyte-derived pro-inflammatory molecules like leptin, visfatin, and resistin, but also reduced levels of anti-inflammatory adiponectin were described [2,4-6], the latter down-regulating innate immune response in cells that express the adiponectin receptor, like macrophages, and monocytes [7-9]. Besides the adipocyte itself, the AT macrophage as a major cell type of the AT of about 10%, is contributing largely to a pro-inflammatory situation, secreting increased levels of cytokines like TNFα, IL-6, C-reactive protein, monocyte chemoattractant protein 1 (MCP-1), and leptin as well [4]. A pre- or manifested obese state of a patient may be a significant predisposition affecting inflammatory responses to immune challenging stimuli.

Epidemiologic studies support a relationship between the AT and the lung, indicating extreme weight loss or gain to have been associated in the development of lung emphysema and asthma, respectively [10-15].

Systemic proinflammatory conditions such as overweight and obesity are discussed to increase the susceptibility to adverse health effects of air pollution. In this context it is believed that pulmonary oxidative stress, resulting from inhaled particulate matter (PM) can lead to pulmonary and systemic inflammation, and subsequently to an increased cardiovascular risk. The fine PM fraction - the fraction with an aerodynamic diameter ≤ 2.5 μm (PM2.5) - is due to its most effective deposition in the distal parts of the lungs regarded as the most hazardous fraction of urban ambient PM. Epidemiological studies reported that relative to people of normal weight, overweight or obese are at increased risk of PM related health effects, associated with markers of cardiovascular impairment like reduced hart rate variability, but also with markers of systemic inflammation like increased blood cytokine levels or white blood cell counts [16-18].

Studies on laboratory animals further showed leptin, its primary role being the regulation of appetite [19,20], to affect surfactant production in type II lung epithelial cells protecting the lung from acute injury [21,22]. Also an increased production of pro-inflammatory cytokines in alveolar macrophages resulting in emphysema-like pathogenesis in adiponectin-deficient mice was described and pre-treatment of the alveolar macrophage with adiponectin lead to suppressed secretion of pro-inflammatory cytokines indicating adiponectin as a potent anti-inflammatory molecule [23] and adipokine receptors being expressed in the murine lung already during embryogenesis [24].

According to the described results in literature that obesity locally and systemically can induce a pro-inflammatory environment we used C57BL/6J mice as a model for human obesity, induced by feeding a high caloric diet [25,26]. We tested the hypothesis, whether exposing mice to the diet challenge for a short period of six weeks will result in higher body mass gain, changes in body composition, and whether early diet-induced changes on body parameters result in a basal pro-inflammatory state in the lung alveolar compartment and blood systemic level by investigating cellular composition and changes in molecular inflammatory factors. Finally, we wanted to clarify, whether diet-induced changes will affect/exaggerate the particle-triggered inflammatory response in the alveolar compartment on a cellular and molecular level. Therefore, carbon-nanoparticle (CNP) exposure was chosen as a surrogate for urban air pollution by combustion derived nanoparticles and as an enormously increasing anthropogenic source of indoor particulate matter with more than 10 million tones produced per year [27]. But regardless of CNP ancestry, this sub-100 nm scaled particle class in general gained toxicological interest due to the factors small dimensions, large surface area, and deposition efficiency in the lung, considering it to be important in driving adverse health effects linked to respiratory toxicity [14,28,29].

## Results

### Diet-induced effects on body mass and body composition

**Figure 1:**
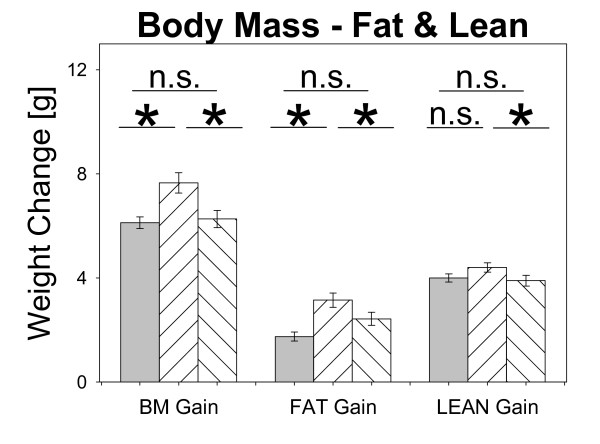
**Body mass (BM), Fat mass and Lean mass gain of animals fed Low Fat (LF; grey bars; n = 25), Cafeteria (CA; coarse left bars; n = 23) or High Fat (HF; coarse right bars; n = 26) diet for six weeks, respectively**. *Statistics*: *post-hoc Holm-Sidak method *(*P < 0.05) after significant *One-Way ANOVA *(diet: F = 6.604; *P = 0.002).

Feeding animals for six weeks with diets of different caloric content (Additional File 1, Table S1) resulted in changes in body mass as well as body composition. All animals gained body mass during the trial, but only Cafeteria (CA; n = 23) animals, which were fed the diet with intermediate caloric content, were significantly heavier than Low Fat (LF; n = 25, + 1.2 g) and High Fat (HF; n = 26, + 1.1 g) animals (Figure 1, Additional File 1 Table S2), respectively. Further, CA and also HF animals showed a shift in body composition with fat mass being slightly increased compared to LF mice. Gain in lean mass of CA was only slight compared to mice fed HF diet (Figure 1). All together, results point towards a pre-obese state especially in Cafeteria diet fed mice after a only short term six week feeding period whereas HF mice did not gain as much weight but also showed increased body fat when compared with LF mice. The number of animals used and details on group settings are provided in Table 1.

**Table 1:** Group settings: animals were fed Low Fat (LF), Cafeteria (CA), or High Fat diet for six weeks, respectively, or were left undisturbed (home cage control, HCC), and were investigated 24 h after intratracheal challenge with Printex 90 carbon-nanoparticles (CNP) or water instillation (SHAM).

Diet fed (6 weeks)	Home Cage Control (HCC)	H_2_0 instilled Controls (SHAM)	CNP Instillation-24 h (CNP)
Low Fat (LF)	8	8	9

Cafeteria (CA)	8	7	8

High Fat (HF)	9	8	9

### Diet and treatment induced effects on BAL and Blood Cell Differentials Bronchoalveolar Lavage (BAL) cell analysis, protein and lactate dehydrogenase

Bronchoalveolar lavage (BAL) volumes gained from age-matched animals fed LF, CA, and HF diet under untreated home cage control conditions (HCC), SHAM and CNP conditions did not reveal significant differences and allowed an adequate comparison of BAL cell differentials.

**Figure 2:**
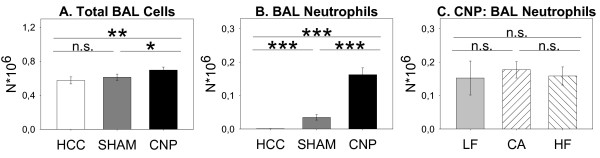
**Total BAL cells (A) and BAL neutrophil counts (B) of untreated home cage controls (HCC; n = 25), SHAM-exposed (SHAM; n = 23), and CNP-instilled mice (CNP; n = 26) six weeks after diet challenge, irrespective of diet fed (LF; CA; HF)**. C: BAL neutrophils of CNP-instilled mice, previously fed on Low Fat (LF; grey bars; n = 9), Cafeteria (CA; coarse left bars; n = 8), or High Fat (HF; coarse right bars; n = 9) diet for six weeks, respectively. *Statistics*: *post-hoc MWU *test after significant *Two-Way-ANOVA*.

*Two-Way ANOVA *of cytospin preparations did not reveal differences in total BAL cell numbers and respective macrophage, lymphocyte, plymorphonuclear neutrophil and eosinophil subsets by LF, CA, and HF feeding per se, since the factor *diet *was not significant (data not shown; for statistics please see Table 2a-e). However, *ANOVA *showed a significant influence of *treatment *on BAL cell differentials and *post-hoc MWU *test revealed total BAL cells to be slightly higher due to CNP-instillation - but not due to SHAM-exposure or in untreated HCC animals (Figure 2A). Therefore, results did not provide any hint for a general more pro-inflammatory status in the lung compartment of CA and HF males compared to animals fed on low fat diet (LF), since neutrophil counts were almost absent in untreated HCC groups and SHAM-exposed mice (Figure 2B; Table 2). This finding is in line with earlier investigations revealing lack of inflammatory neutrophils in absences of a lung-specific stimulus [30]. The particle-induced increase in total BAL cell numbers was mainly driven by an enormous influx of neutrophil granulocytes into the alveolar compartment, indicating acute inflammatory response, as expected (Figure 2B). To a small degree higher eosinophilis, but not the macrophage and lymphocyte subset, contributed to overall higher total BAL cell numbers, too (data not shown). The absence of a significant *diet × treatment *interaction term (*ANOVA*; Table 2a-e) and *post-hoc MWU *test neither provided a hint for a difference in the magnitude of inflammatory neutrophil influx between LF, CA, and HF animals by particle challenge (CNP) (Figure 2C) nor with respect to any other BAL cell subset (data not shown).

**Table 2:** *Two-Way ANOVA *of BAL cellular components, BAL protein and BAL lactate dehydrogenase content (LDH) of mice fed 6 weeks on different diets (LF, n = 25; CA, n = 23; HF, n = 26) and challenged by instillation.

Response variables	Predictor variables	*F*	*P*
(a) BAL cell count	diet	0.68	0.51
	treatment	4.83	*0.011
	diet × treatment	2.01	0.10
	
(b) BAL macrophages	diet	0.36	0.69
	treatment	0.32	0.73
	diet × treatment	1.17	0.33
	
(c) BAL lymphocytes	diet	1.36	0.26
	treatment	3.63	*0.032
	diet × treatment	0.61	0.66
	
(d) BAL neutrophils	diet	0.65	0.53
	treatment	54.06	*** < 0.001
	diet × treatment	0.19	0.94
	
(e) BAL eosinophils	diet	1.24	0.29
	treatment	3.80	*0.027
	diet × treatment	1.22	0.31
	
(f) BAL Protein	diet	8.57	*** < 0.001
	treatment	3.43	*0.038
	diet × treatment	0.08	0.99
	
(g) BAL LDH	diet	5.24	**0.008
	treatment	0.94	0.40
	diet × treatment	0.43	0.79

**Figure 3:**
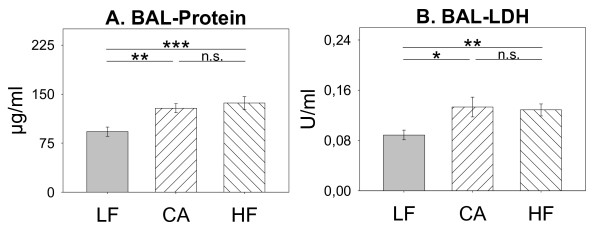
**BAL protein (A) and BAL lactate dehydrogenase (LDH) content (B) of mice fed Low Fat (LF; grey bars; n = 25), Cafeteria (CA; coarse left bars; n = 23), or High Fat (HF; coarse right bars; n = 26) diet for six weeks, respectively, irrespective of treatment (HCC; SHAM; CNP)**. *Statistics*: *post-hoc MWU *test after significant *Two-Way-ANOVA*.

In contrast to an absence of differences in the BAL cellular composition by diet-feeding, CA and HF diet (ANOVA: *diet *significant) resulted in significantly higher concentrations of the intracellular enzyme lactate dehydrogenase (LDH) and total protein content in BAL fluid in comparison to mice fed on caloric low fat diet (LF) (Figure 3A,B; Table 2f). These higher protein concentrations in the alveolar lumen indicated a damage of the barrier function of the alveolar-capillary membrane and were a sign for increased lung injury. Instillation of CNPs in general increased BAL protein content (data not shown; *ANOVA: treatment *significant, Table 2f), irrespective of the diet fed (*ANOVA: treatment *x *diet *not significant, Table 2f). In line, higher LDH further points towards diet-induced increased cell membrane damage and necrotic cell death in BAL cells of CA and HF mice from a biological point of view. Differences by instillation (*ANOVA: treatment *not significant) were not observed.

### Hematological analysis

**Figure 4:**
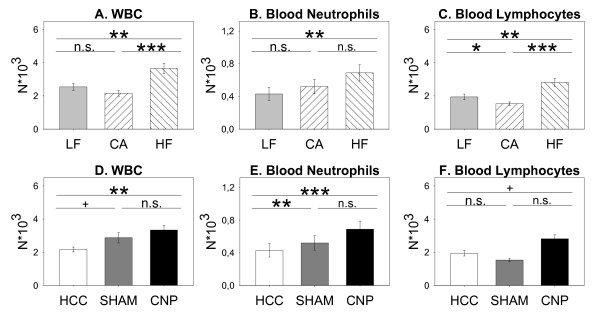
**Diet-induced effects on white blood cells (WBC) (A), blood neutrophils (B), and blood lymphocytes (C) of mice fed Low Fat (LF; grey bars; n = 25), Cafeteria (CA; coarse left bars; n = 23), or High Fat (HF; coarse right bars; n = 26) diet for six weeks, respectively**. Treatment-induced effects on respective parameters by SHAM or CNP exposure in comparison to untreaeted home cage controls (HCC) (D-F). *Statistics*: *post-hoc MWU *test after significant *Two-Way-ANOVA*.

On the blood systemic level, six weeks feeding period (*diet*) resulted in significantly higher white blood cell (WBC) numbers in HF, but not CA animals, compared to LF animals (Table 3a-e; Figure 4A). This leukocytosis in HF mice was mainly due to a significantly higher number of lymphocytes (Table 3 Figure 4C), and to lower degree due to higher neutrophil counts (Table 3 Figure 4B), Moreover, *ANOVA *provided a significant *treatment *effect on total leukocyte numbers (WBC), lymphocytes, and the neutrophil subset (Table 3) with particle instillation resulting in higher cell numbers, respectively (Figure 4D-F) compared to HCC groups, being statistically significant or at least different at trend. Neither *diet*- nor *treatment*-induced differences were observed among the monocyte (*post-hoc MWU*) and the eosinophil subset (*ANOVA*: n.s.; data not shown), respectively. Accordingly and in line with the results on BAL cell differentials, particle instillation increased blood neutrophils, indicating an activation of the blood system on top to a slight but general more pro-inflammatory blood systemic status in HF animals by diet feeding (Figure 4B).

**Table 3:** *Two-Way ANOVA *of blood cell differentials of mice fed 6 weeks on different diets (LF, n = 25; CA, n = 23; HF, n = 26) and challenged by instillation.

Response variables	Predictor variables	*F*	*P*
(a) White blood cells	diet	13.31	*** < 0.001
	treatment	7.48	**0.001
	diet × treatment	0.49	0.75
	
(b) Blood neutrophils	diet	5.16	**0.008
	treatment	12.31	*** < 0.001
	diet × treatment	0.58	0.68
	
(c) Blood lymphocytes	diet	16.09	*** < 0.001
	treatment	2.51	^+^0.089
	diet × treatment	1.10	0.36
	
(d) Blood monocytes	diet	0.90	0.41
	treatment	11.58	*** < 0.001
	diet × treatment	0.61	0.66
	
(e) Blood eosinophils	diet	2.00	0.14
	treatment	1.53	0.22
	diet × treatment	0.87	0.49

### BAL and Blood Inflammatory Marker Proteins - Adipokine & Cytokine Levels

In order to get further insight into the molecular events triggered by diet-feeding per se and in combination with the particle challenge, BAL and blood serum were examined. A set of known adipokine marker proteins (Table 4) as well as some selected protein markers involved in lung inflammatory reactions were checked (Table 5).

**Table 4:** Two-Way ANOVA/*MWU *test of protein markers in BAL fluid of mice fed 6 weeks on different diets (LF, n = 25; CA, n = 23; HF, n = 26) and challenged by instillation.

Response variables	Predictor variables	*F*	*P*
(a) BAL Leptin	diet	5.08	**0.009
	treatment	0.12	0.89
	diet × treatment	1.09	0.37
	
(b) BAL Adiponectin	diet	1.82	0.17
	treatment	0.32	0.73
	diet × treatment	0.87	0.49
	
(c) BAL Fibrinogen	diet	2.14	0.13
	treatment	5.43	**0.007
	diet × treatment	1.81	0.14
	
(d) BAL PAI-1	diet	0.39	0.68
	treatment	17.98	*** < 0.001
	diet × treatment	0.45	0.77
	
(e) BAL IL-1α	Diet (*MWU*)	n.s.	n.s.
	Treatment (*MWU*)	n.s.	n.s.
			
	
(f) BAL IL-1β	diet	2.14	0.13
	treatment	21.35	*** < 0.001
	diet × treatment	0.36	0.84
	
(g) BAL IL-4	diet (*MWU*)	n.s.	n.s.
	treatment (*MWU*)	n.s.	n.s.
			
	
(h) BAL IL-5	diet	0.52	0.59
	treatment	84.73	*** < 0.001
	diet × treatment	1.12	0.36
	
(i) BAL IL-6	diet	1.00	0.37
	treatment	83.20	*** < 0.001
	diet × treatment	0.21	*** < 0.001
	
(j) BAL IL-10	diet (*MWU*)	n.s.	n.s.
	treatment (*MWU*)	n.s.	n.s.
			
	
(k) BAL IL-12(p40)	diet	0.14	0.87
	treatment	20.35	*** < 0.001
	diet × treatment	0.79	0.54
	
(l) BAL IL-12(p70)	diet (*MWU*)	n.s.	n.s.
	treatment (*MWU*)	n.s.	n.s.
			
	
(m) BAL IFNγ	diet	7.85	*** < 0.001
	treatment	0.32	0.73
	diet × treatment	2.82	*0.033
	
(n) BAL TNFα	diet (*MWU*)	n.s.	n.s.
	treatment (*MWU*)	n.s.	n.s.
			
	
(o) BAL G-CSF	diet	0.80	0.45
	treatment	149.03	*** < 0.001
	diet × treatment	0.90	0.47
	
(p) BAL CXCL1	diet	3.85	*0.027
	treatment	54.16	*** < 0.001
	diet × treatment	3.20	*0.019
	
(q) BAL CCL5	diet (*MWU*)	n.s.	n.s.
	treatment (*MWU*)	n.s.	n.s.
			
	
(r) BAL MIP-2	diet	3.95	*0.024
	treatment	44.64	*** < 0.001
	diet × treatment	2.85	*0.031

**Table 5:** Two-Way ANOVA of protein markers in blood serum of mice fed 6 weeks on different diets (LF, n = 25; CA, n = 23; HF, n = 26) and challenged by instillation.

Response variables	Predictor variables	*F*	*P*
(a) Serum Leptin	diet	0.42	0.66
	treatment	2.14	0.13
	diet × treatment	0.35	0.84
	
(b) Serum Adiponectin	diet	3.21	*0.047
	treatment	0.66	0.52
	diet × treatment	0.58	0.68
	
(c) Serum Fibrinogen	diet	3.08	^+^0.052
	treatment	4.33	*0.017
	diet × treatment	0.07	0.99
	
(d) Serum PAI-1	diet	0.55	0.58
	treatment	1.87	0.16
	diet × treatment	1.20	0.32
	
(e) Serum IL-1α	diet	2.21	0.12
	treatment	0.80	0.45
	diet × treatment	0.59	0.67
	
(f) Serum IL-1β	diet	1.53	0.22
	treatment	2.56	^+^0.085
	diet × treatment	0.43	0.78
	
(g) Serum IL-4	diet	1.14	0.33
	treatment	0.61	0.54
	diet × treatment	1.31	0.28
	
(h) Serum IL-5	diet	0.57	0.57
	treatment	13.76	*** < 0.001
	diet × treatment	0.15	0.96
	
(i) Serum IL-6	diet	0.12	0.89
	treatment	6.02	**0.004
	diet × treatment	0.59	0.67
	
(j) Serum IL-10	diet	1.48	0.24
	treatment	2.36	0.10
	diet × treatment	0.85	0.50
	
(k) Serum IL-12(p40)	diet	1.81	0.17
	treatment	3.42	*0.038
	diet × treatment	0.47	0.76
	
(l) Serum IL-12(p70)	diet	0.81	0.45
	treatment	1.61	0.21
	diet × treatment	0.76	0.56
	
(m) Serum IFNγ	diet	0.16	0.85
	treatment	2.54	^+^0.086
	diet × treatment	0.60	0.66
	
(n) Serum TNFα	diet	0.93	0.40
	treatment	1.29	0.28
	diet × treatment	0.33	0.86
	
(o) Serum G-CSF	diet	1.88	0.16
	treatment	4.45	*0.015
	diet × treatment	1.24	0.30
	
(p) Serum CXCL1	diet	4.09	*0.021
	treatment	2.04	0.14
	diet × treatment	1.16	0.34
	
(q) Serum CCL5	diet	5.09	**0.009
	treatment	0.74	0.48
	diet × treatment	0.36	0.84
	
(r) Serum MIP-2	diet	2.10	0.13
	treatment	3.37	*0.040
	diet × treatment	1.32	0.27

**Figure 5:**
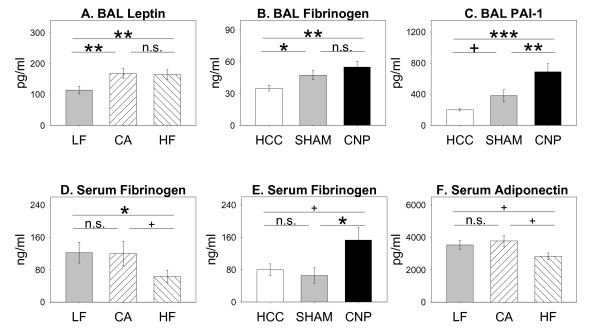
**Diet- and treatment induced effects on adipokine concentrations in BAL (A-C) and blood serum (D-F)**. Mice fed Low Fat (LF; grey bars; n = 25), Cafeteria (CA; coarse left bars; n = 23), or High Fat (HF; coarse right bars; n = 26) diet for six weeks, respectively. Untreated home cage controls (HCC; white bars; n = 25), SHAM exposed (SHAM; grey bars; n = 23), and CNP instilled mice (CNP; black bars; n = 26). *Statistics*: *post-hoc MWU *test after significant *Two-Way-ANOVA*.

Among adipocyte-derived marker proteins the analysis of pro-inflammatory BAL Leptin revealed significant differences caused by diet feeding among groups with higher concentrations in BAL of CA and HF animals (Table 4, Figure 5A), the diets with higher caloric content, compared to LF animals but not being further influenced by *treatment *(ANOVA: *treatment*: n.s.). In contrast, CNP instillation and to lower degree also SHAM exposure elevated BAL fibrinogen concentrations and the adipocyte-derived fibrinolytic factor plasminogen-activator inhibitor 1 (PAI-1) (Figure 5B,C), whereas diet-induced differences were absent. BAL anti-inflamamtory Adiponectin levels were neither affected by diet nor by instillation challenge (data not shown). Interestingly, the higher diet-induced BAL Leptin values were not reflected on a blood systemic level, nor, there was a treatment-induced difference in BAL and blood (data not shown), as in contrast was observed for BAL PAI-1 and fibrinogen. Remarkably, concentrations of anti-inflammatory Adiponectin in serum were reduced only in HF fed animals - but not CA fed mice which showed changes in body composition with higher fat mass gain - therefore pointing towards a more pro-inflammatory blood systemic situation in HF animals although diet-induced effects were absent in this group (Figure 5F). In line, HF diet led to lower serum Fibrinogen compared to CA and LF animals, although CNP instillation in general resulted in significantly increased parameter values (Figure 5D,E).

**Figure 6:**
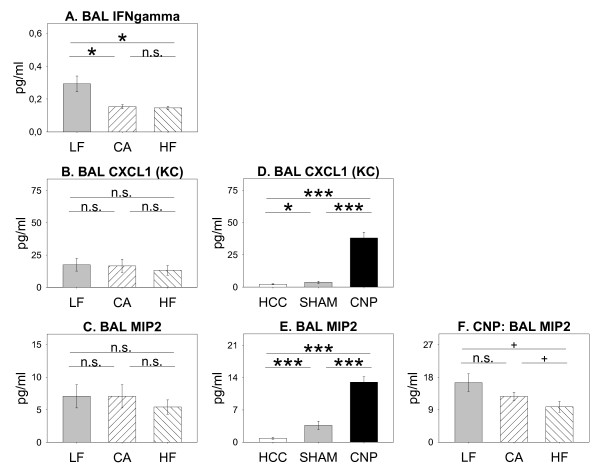
**Diet- and treatment induced effects on cytokine concentrations in BAL (A-F)**. Mice fed Low Fat (LF; grey bars; n = 25), Cafeteria (CA; coarse left bars; n = 23), or High Fat (HF; coarse right bars; n = 26) diet for six weeks, respectively. Untreated home cage controls (HCC; white bars; n = 25), SHAM exposed (SHAM; grey bars; n = 23), and CNP instilled mice (CNP; black bars; n = 26). *Statistics*: *post-hoc MWU *test after significant *Two-Way-ANOVA*.

**Figure 7:**
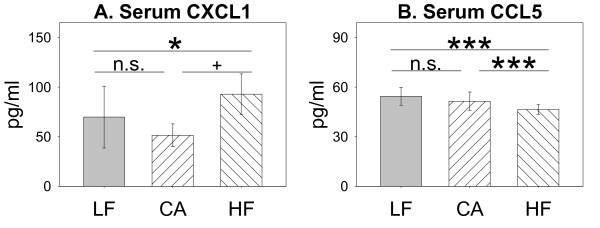
**Diet-induced effects on serum cytokine concentrations (A, B)**. Mice fed Low Fat (LF; grey bars; n = 25), Cafeteria (CA; coarse left bars; n = 23), or High Fat (HF; coarse right bars; n = 26) diet for six weeks, respectively. *Statistics*: *post-hoc MWU *test after significant *Two-Way-ANOVA*.

Analysis of investigated inflammatory cytokines further revealed an absence of diet- and treatment-induced effects on the concentrations of IL-1α, IL-4, IL-10, IL-12(p70), and TNFα, both, in BAL fluid and blood serum (data not shown). Additionally, BAL CCL5 and serum IFNγ were unaffected (data not shown). *ANOVA *(*diet *significant) indicated diet-induced differences in cytokine levels in BAL compartment for IFNγ, CXCL1 and MIP2/CXCL2 (Figure 6A-C); however *post-hoc MWU *test only confirmed diet-induced lower macrophage activating factor IFNγ in HF and CA group to be significant. Also serum CCL5 was reduced by HF diet, while CXCL1 concentrations (Figure 7A, B) were increased. In BAL, values for CXCL1 and MIP2/CXCL2 (Figure 6D,E), but not IFNγ, (data not shown) were increased by particle treatment. Particle induced increase was further observed for BAL and serum IL-1β, IL-5, IL-6, IL-12(p40), and G-CSF without being affected by diet-feeding per se (Additional File 1, Figure S1). HF animals at trend further showed MIP-2 concentrations to be less increased by particle treatment compared to LF and CA animals (Figure 6F).

## Discussion

In this study a combined diet-particle challenge model was used. The purpose of this study on the one hand was to elicit the impact of short term feeding of a high caloric carbohydrate- or fat-rich diet, respectively, on body mass gain and body composition. Moreover, we elucidated whether diets specifically affected the basal cellular and molecular status of the blood and the alveolar compartment in terms of signs for pre-obesity-triggered pro-inflammatory differences. Finally, we investigated the impact of the different diets on the inflammatory response in a well-defined carbon-nanoparticle-triggered lung inflammation model [30].

### Diet-induced body mass changes - distinct effects on body composition & systemic and alveolar pro-inflammatory status

Interestingly, we showed here that not the high fat diet (HF), with highest caloric content, but a carbohydrate-rich and calorically intermediate Cafeteria diet (CA) resulted in marked body mass gain and changes in body composition, as indicated by increased fat mass, compared to mice fed on a low fat control diet or mice fed HF. Therefore, not primarily the caloric content but the composition and flavour of the diet was the more important issue inducing a pre-obese state in our animal model. The characteristics of diet induced obesity triggered by a cafeteria diet, as compared to a regular HF diet is currently under investigation. It can be assumed that varying flavours (e.g. chocolate) stimulate hyperphagia and over-consumption of energy. To our surprise and in contrast, differences in systemic blood cellular immunity were not affected by CA diet and rather High Fat diet resulted in a marked activation of the immune system as indicated by an increase in lymphocyte counts and also slightly higher numbers of inflammatory neutrophils, pointing towards slightly increased general inflammatory status. However both, CA and HF diet resulted in higher total BAL protein and BAL lactate dehydrogenase content, indicating a disturbance in the alveolar compartment, and possibly even increased cell necrosis and a loss of the alveolar-capillary membrane in its function as a barrier, in general allowing selective influx of molecular and cellular components into the alveolar space. Regardless, both high caloric diets did not result in an increased inflammatory status in the alveolar region as far as inflammatory cell influx is concerned. Further, while lowering IFNγ concentrations in BAL, significantly increased pro-inflammatory Leptin was found in BAL as well, which may be considered as a hint towards pro-inflammation from a molecular point of view, compared to mice fed LF diet. A higher pro-inflammatory status is further supported by reduced concentrations of anti-inflammatory adiponectin in the serum of HF mice only, but not CA fed mice, however again, the latter not affecting blood cellular composition or magnitude of cell numbers and may point towards distinct counterbalancing effects depending on the diet fed. All together the results clearly show that the only short term six weeks feeding period was well chosen to investigate the early events taking place at the onset of diet-induced metabolic changes and its impact on cellular and molecular components in the alveolar and the blood systemic compartment before systems derail.

### Carbon-nanoparticle-triggered inflammatory response in diet-fed mice

As expected CNP challenge resulted in a dramatic recruitment of inflammatory neutrophils into the alveolar compartment [30], however did not provide differences in the magnitude among diet-fed groups. Also several inflammatory and risk marker proteins concomitantly were elevated in BAL and serum, like the granulocyte colony stimulating factor (G-CSF), monocyte/macrophage-derived inflammatory proteins IL-1β and IL-12(p40), eosinophil chemo-attractant IL-5, and the inflammatory and cardiovascular risk factors IL-6 and adipocyte- or lung epithelial derived Fibrinogen [31,32], all together showing effectiveness of the challenge itself. Interestingly, although particle challenge in general increased PAI-1, CXCL1, and MIP2/CXCL2 concentrations in BAL, HF animals revealed at least at trend lower BAL macrophage inflammatory protein 2 (MIP2) values in comparison to LF and CA mice, respectively, and therefore may indicate a lower degree of macrophage pro-inflammatory activation which in turn partially may be responsible for an absence in the extent of neutrophil influx among groups. Synergistically, also reduced macrophage-activating BAL IFNγ concentrations, found in both, HF and CA mice, may have contributed to an absence of neutrophil differences in the alveolar compartment. The absence of a stronger cellular inflammatory response in CA and HF mice, despite an increased BAL Leptin content, represents an interesting finding, since Leptin resistance was shown to reduce hyperoxia-induced lung inflammation and to protect from lung fibrosis [33,34]. Accordingly, the increased BAL Leptin found in the present study would have been expected to increase the inflammatory response in CA and HF mice. In this context, a recent study showed exogenous Leptin administration to reduce the cellular inflammatory response in a chronic COPD model [35]. Therefore, in the present study, either Leptin in BAL acted anti-inflammatory and prevented an increased particle related neutrophil influx in CA and HF mice, or, if BAL Leptin action was pro-inflamamtory, then other molecular factors may have counterbalanced, like the reduced MIP2 and IFNγ concentrations in BAL, which predominantly can be dedicated to be of alveolar macrophage origin. Seen from this angle, the differences in HF mice, regarding their inflammatory state in the blood system appear more to be affected by the reduced anti-inflammatory serum Adiponectin levels.

## Conclusion

All together our results provide insight into the changes of pro- and anti-inflammatory marker proteins caused by feeding diets of different caloric content and composition. The data show, that although the diet-challenge already was efficient in driving pro-inflammatory systemic events, extended feeding periods are necessary to generate an increased susceptibility to particle-induced lung inflammation.

## Methods

### Animals and Experimental Setup

Male C57BL/6J mice were received (Charles River, 97633 Sulzfeld, Germany) at an age of four weeks. Upon entry into our animal facility, animals were kept in isolated ventilated cages (IVC-Racks; BioZone, Margate, UK) supplied with filtered air, in a 12-hr light/12-hr dark cycle (lights on from 06:00 - 18:00) and allowed to adapt to conditions for approximately three weeks (19-20 days). Food (standard chow) and water were available *ad libitum*. After acclimatisation period animals were divided into three weight-matched groups and fed different diets (SSniff Spezialdiäten GmbH, 59494 Soest, Germany) for a period of six weeks, respectively. One group remained on a standard low fat diet (Low Fat; LF, gross energy content 16.27 kJ/g dry mass; n = 25) isocaloric to the chow fed during acclimatisation period, the second group (n = 23) was fed a carbohydrate-rich Cafeteria-diet (Cafeteria; CA; sodium/rich biscuit/sugar; gross energy content 18.94 kJ/g dry mass). The three different cafeteria diets were mixed in equal proportions. The third group (n = 26) was fed a high-caloric fat-diet (High Fat; HF; gross energy content 23.54 kJ/g dry mass). All procedures for animal handling and experiments were performed in accordance with protocols approved by the Regierung von Oberbayern (District Government of Upper Bavaria). Experiments were performed at the German Mouse Clinic phenotyping platform (GMC) [36,37].

### Body Mass and Body Composition

Body mass of animals was monitored at the day of delivery (4 weeks old) and the day the diet challenge started (7 weeks old). Further body mass development was monitored in weekly intervals until the end of six weeks' feeding period. Body composition was determined at delivery; at the beginning of the diet challenge as well as every second week during the diet-feeding (after week 2, 4, 6, respectively) using a whole animal body composition analyzer (Minispec Bruker, Ettlingen Germany) based on Time Domain Nuclear Magnetic Resonance (TD-NMR) which provides a precise method for the measurement of lean tissue and body fat in live mice without anaesthesia. To conduct the measurement single mice were placed in a plastic restrainer that was introduced into the magnet of the scanner for up to 5 minutes to collect data. Data acquisition was based on a calibration using dissected lean muscle and fat tissue. For the statistical analysis, a linear regression model was applied including diet as main factor and body mass as covariate to adjust for body mass differences.

### Particle Challenge Design and Group Setup

Particle challenge was done directly after six weeks' feeding period in the 13 weeks old animals. In a counterbalanced system, animals of all three groups (LF, CA, HF) were either instilled with an aqueous suspension of Printex 90 carbon-nanoparticles (CNP) as previously described [38] (zeta potential: 33 mV; agglomerate diameter in suspension: 0.17 μm), or pyrogene-free distilled water (SHAM exposed) respectively or were left undisturbed and served as controls (Home Cage Control; HCC). Printex 90 was chosen as frequently used commercially available pigment black (Degussa, Frankfurt, Germany) (diameter [nm]: 14; organic content [%]: 1; surface area [m^2^/g]: 272); as characterized earlier [30,39]. For details on group setup and sample size, see Table 1.

Prior to instillation, mice were anesthetized by intraperitoneal injection of a mixture of Medetomidin (0.5 mg/kg body mass), Midazolam (5.0 mg/kg body mass) and Fentanyl (0.05 mg/kg body mass). The animals were then intubated by a nonsurgical technique (Brown et al. 1999). Using a cannula inserted 10 mm into the trachea, a suspension containing 20 μg CNPs particles, respectively, in 50 μL pyrogene-free distilled water was instilled, followed by 100 μL air; the suspension of poorly soluble CNPs was sonicated on ice for 1 min prior to instillation, using a SonoPlus HD70 (Bachofer, Berlin, Germany) at a moderate energy of 20 W resulting in a mean agglomerate size of 0.17 μm (Zetasizer Nano ZS, Malvern Instruments, Herrenberg, Germany). SHAM animals were instilled 50 μL pyrogene-free distilled water only [40]. After instillation animals were antagonized by subcutaneous injection of a mixture of Atipamezol (2.5 mg/kg body mass), Flumazenil (0.5 mg/kg body mass) and Naloxon (1.2 mg/kg body mass) to guarantee their awakening and well-being. Animals were treated humanely and with regard for alleviation of suffering; experimental protocols were reviewed and approved by the Bavarian Animal Research Authority.

### Blood, Serum, and Bronchoalveolar Lavage (BAL) sampling

Twenty-four hours after instillation, mice were anesthetized by intraperitoneal injection of a mixture of xylazine (4.1 mg/kg body weight) and ketamine (188.3 mg/kg body weight) and killed by exsanguination. Therefore, blood was drawn from the retroorbital plexus by a capillary and collected a.) in EDTA covered tubes (*Sarstedt*) for haematological analysis (ADVIA Hematology Systems (Bayer Diagnostics) and b.) non EDTA-covered tubes to gain blood serum. Subsequently BAL was performed by cannulating the trachea and infusing the lungs 10 times with 1.0 mL PBS without calcium and magnesium, as described previously [40]. The BAL fluids from lavages 1 and 2 and from lavages 3-10 were pooled and centrifuged (425 *g*, 20 min at room temperature). The cell-free supernatant from lavages 1 and 2 were pooled and used for biochemical measurements such as lactate dehydrogenase (LDH), total protein, and cytokine concentration. The cell pellet was resuspended in 1 mL RPMI 1640 medium (BioChrome, Berlin, Germany) and supplemented with 10% foetal calf serum (Seromed, Berlin, Germany); the number of living cells was determined by the trypan blue exclusion method. We performed cell differentials on the cytocentrifuge preparations (May-Grünwald-Giemsa staining; 2 × 200 cells counted) and the number of polymorphonuclear leukocytes (PMNs) was used as a marker of inflammation.

### BAL: Total Protein Content and Lactate Dehydrogenase (LDH) Assay

Total BAL protein content was determined spectrophotometrically with an ELISA reader (Labsystems iEMSReader MF, Helsinki, Finland) at 620 nm, applying the Bio-Rad Protein Assay Dye Reagent (no. 500-0006; BioRad, Munich, Germany), as a potential biological marker for pulmonary capillary leakage and lung injury [31]. 5 μl BAL fluid/mouse was used for analysis.

For detection of the cytosolic enzyme lactate dehydrogenase (LDH) (U/ml), characteristic for membrane damaging effects, the Cytotoxicity Detection Kit (Roche Diagnostics, Germany) was used according to the manufacturer's instructions. LDH concentration in the BAL fluid (30 μl) was spectrophotometrically determined with an ELISA reader (Labsystems iEMS Reader MF, Helsinki, Finland) at a wavelength of 492 nm.

### Cytokine and Adipokine Detection (Multiplexed immunoassays)

BAL fluid and blood serum adipokine concentrations were investigated using a Luminex xMAP system (Milliplex, mouse CVD Panel 2 and mouse adipocyte Panel, Millipore Corporation, 290 Concord Road, Billeria, MA 01821, USA) to simultaneously detect the concentration for the 4 following adipokine analytes: fibrinogen, leptin, adiponectin, PAI-1. Measurement was performed according to the manufacturer's instructions.

Accordingly to adipokine analysis, also cytokine/chemokine concentrations were investigated in BAL fluid and blood serum using Bio-Plex Pro Mouse 14Plex Cytokine Panel, Bio-Rad Laboratories, 2000 Alfred Nobel Drive, Hercules CA 94547, USA).

Simultaneously the following 14 cytokines/chemokines were investigated in cell-free BAL fluid (50 μl) or blood serum (15 μl serum + 35 μl sample diluent). Analytes were as follows: IL-1α, IL-1β, IL-4, IL-5, IL-6, IL-10, IL-12(p40), IL-12(p70), IFN-γ, TNF-α, CXCL2 (MIP2), G-CSF, CCL5 (RANTES), CXCL1 (KC).

For all measurements the mean fluorescence intensity (MFI) was detected by the Multiplex plate reader (Luminex System, Bio-Rad Laboratories, Germany). For each sample a minimum of 50 beads per region were analyzed. Multiplex plate reader software (Bioplex Manager, Version 4.1.1) was used to capture raw data (MFI). For data analysis, a four-parameter logistic curve fit was applied to each standard curve and sample.

### Statistics

We tested the effects of the two factors *diet *(3 levels: Low Fat diet (LF), Cafeteria diet (CA), High Fat diet (HF)) and *treatment *(3 levels: untreated home cage control (HCC), water-instilled SHAM group at 24 h (SHAM), and CNP instillation after 24 h (CNP)) on different response variables, as shown in Tables 2, 3, 4, 5 (*Two-Way ANOVA*). We included the interaction of the two factors (*diet *x *treatment*) in order to test whether the treatment showed differential effects within the different diet groups. If not statistically significant, the interaction term was reduced and the model was re-calculated. Response variables, which deviated from the normal distribution, were log-, or square-root-transformed. Normality of the model residuals was checked visually by normal probability plots and with the Shapiro-Wilk test, and we assured the homogeneity of variances and goodness of fit by plotting residuals versus fitted values and by the Levene test [41]. Post-hoc comparisons were conducted with the Mann-Withney *U *test (*MWU*). In cases transformation of data failed to reach normal distribution *MWU *test was performed as statistical test. All statistical analyses were done using the software SPSS 11.0 (SPSS Inc., Chicago, IL).

Body mass, lean and fat mass data accordingly were tested by *One-Way ANOVA *for the factor *diet *only (Figure 1). *Holm-Sidak method *was used for post-hoc comparison between diet-fed groups.

All data are expressed as mean ± SEM. Within graphs significant *P*-values are shown by asterisks (*P < 0.050, **P < 0.010, ***P < 0.001) and trends are indicated by plus symbol (^+^*P *< 0.10).

## Competing interests

The authors declare that they have no competing interests.

## Authors' contributions

AG, JR and TS conceived and designed the experiments. AG and JR performed the experiments. AG, JR, and HR analyzed the data. AG, JR, HGR, HF, VG, MHA, MK, and TS wrote the manuscript. All authors read and approved the final manuscript.

## Supplementary Material

Additional file 1 **Additional File **1**, Figure S1 showing treatment-induced effects on cytokine concentrations in BAL fluid and blood serum; Additional File **1**, Tables **1**and **2**, giving information about diet composition and energy content (manufacturer information) of Low Fat, (LF), Cafeteria (CA), and High Fat (HF) diet and providing overview and values of investigated parameters in LF, CA, and HF animals, respectively**. Click here for file
