# Supplementary material for: Comparison of particle-exposure triggered pulmonary and systemic inflammation in mice fed with three different diets
Source: Part Fibre Toxicol. 2011 Sep 27;8:30. doi: 10.1186/1743-8977-8-30 (PMC3197490; doi:10.1186/1743-8977-8-30)
Supplement: Additional file 1 — , Figure S1 showing treatment-induced effects on cytokine concentrations in BAL fluid and blood serum; Additional File 1, Tables 1and 2, giving information about diet composition and energy content (manufacturer information) of Low Fat, (LF), Cafeteria (CA), and High Fat (HF) diet and providing overview and values of investigated parameters in LF, CA, and HF animals, respectively. [file 1743-8977-8-30-S1.PDF]

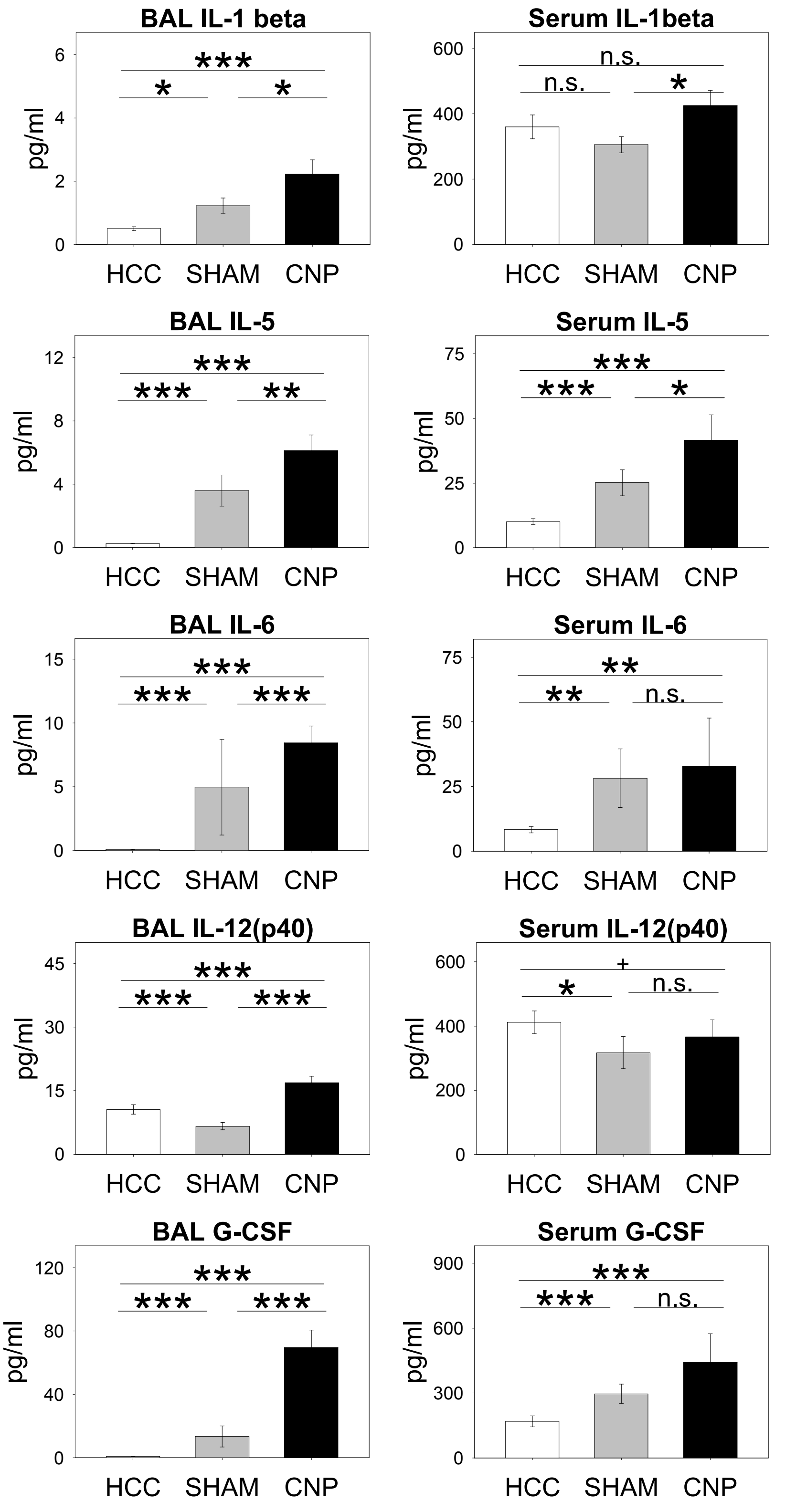

Additional Figure S1:

Treatment-induced effects on cytokine concentrations in BAL fluid and blood serum. Untreated home cage controls (HCC; white bars; n=25), SHAM exposed (SHAM; grey bars;

n=23), and CNP instilled mice (CNP; black bars; n=26). *Statistics*: *post-hoc MWU* test after significant *Two-Way-ANOVA*.

Additional Table S1:

Diet composition and energy content (manufacturer information) of Low Fat, (LF), Cafeteria (CA), and High Fat (HF) diet.

	Item No.<br>Name	S0372-<br>E022x+<br>sodium	S0372-<br>E024x+rich<br>biscuit	S0372-<br>E026x+<br>sugar	E15741-347<br>D12492	E15000<br>Control
		Cafeteria Diets (CA)			High Fat<br>Diet (HF)	Low Fat<br>Diet(LF)
Raw material	Casein	19.00	20.6	20.00	27.69	24.00
	Peanut meal. roasted. Salted	5.00	-	-	-	-
	Corn starch mod.	18.00	32.00	5.00	-	49.80
	Maltodextrin	18.10	5.00	6.40	15.80	-
	Sucrose	12.00	12.30	36.00	8.00	10.00
	Cellulose	4.60	5.00	4.00	6.00	5.00
	Vitamin premix	1.20	1.20	1.20	1.20	1.00
	Mineral/trace elements	6.00	6.00	6.00	6.10	6.00
	L-Cystine	0.25	0.20	0.20	0.35	-
	L-Threonine	0.15	0.15	0.15	-	-
	Choline chloride	0.20	0.20	0.20	0.25	0.20
	Salt (NaCl)	0.89	0.24	0.24	0.10	-
	Butylhydroxytoluol	0.01	0.01	0.01	0.01	-
	Butter fat	5.00	9.00	-	-	-
	Beef tallow (premier jus)	9.60	-	6.00	31.00	-
	Soybean oil	-	0.25	-	3.00	4.00
	Coconut fat	-	7.70	-	-	-
	Coca butter	-	-	9.85	-	-
	Coca powder	-	-	4.60	-	-
	Banana flavour	-	0.10	-	-	-
	Chocolata flavour	-	-	0.10	-	-
Compo sition	Crude Proteine [%]	18.2	18.1	18.4	24.1	20.8
	Crude Fat [%]	17.1	17.0	17.0	34.0	4.2
	Crude Fibre [%]	5.2	5.0	5.4	6.0	5.0
	Crude Ash [%]	6.5	5.8	6.1	6.1	5.6
	Starch [%]	18.9	31.9	6.2	1.1	48.8
	Sugar [%]	12.4	12.8	36.2	8.2	10.8
	Destrins [%]	17.8	4.9	6.3	15.6	-
	N free estracts [%]	49.3	49.9	49.4	27.0	59.4
	Sodium [%]	0.56	0.29	0.29	0.20	0.19
Energy content	GE [MJ/kg]	21.0	21.0	21.1	25.2	18.0
	ME (Atwater) [MJ/kg]	17.9	18.0	18.0	21.4	15.2
	Protein [%]	17	17	17	19	23
	Fat [%]	36	36	36	60	11
	Carbohydrates[%]	47	47	47	21	66

Additional Table S2:

Overview of investigated parameters in LF, CA, and HF animals, respectively. Values are provided as mean±SEM for untreated home cage control (HCC), SHAM exposed, and CNP treated mice.

		Low Fat					
Variable investigated	Unit	HCC Value	SEM	SHAM Value	SEM	CNP Value	SEM
Body Mass Gain	g	6.2	0.3	6.3	0.3	6.0	0.4
Fat Mass Gain	g	4.0	0.3	4.0	0.1	4.0	0.3
Lean Mass Gain	g	1.9	0.3	1.8	0.4	1.6	0.2
BAL Volume	ml	10.9	0.4	10.6	0.4	10.7	0.3
BAL Cells	$N*10^6$	0.6	0.1	0.6	0.0	0.6	0.1
BAL Macrophages	$N*10^6$	0.6	0.1	0.5	0.0	0.4	0.0
BAL Lymphocytes	$N*10^6$	0.011	0.003	0.008	0.002	0.009	0.002
BAL Neutrophils	$N*10^6$	0.0005	0.0003	0.0209	0.0111	0.1524	0.0479
BAL Eosinophils	$N*10^6$	0.0000	0.0000	0.0009	0.0007	0.0026	0.0006
BAL Protein	µg/ml	85.3	14.0	87.5	10.7	105.1	10.0
BAL LDH	U/ml	0.10	0.02	0.09	0.01	0.08	0.01
White Blood Cells	$N*10^3$	2.1	0.3	2.6	0.2	2.9	0.4
Blood Neutrophils	$N*10^3$	0.23	0.07	0.38	0.14	0.65	0.14
Blood Lymphocytes	$N*10^3$	1.77	0.25	1.97	0.11	2.08	0.38
Blood Monocytes	$N*10^3$	0.04	0.01	0.15	0.08	0.15	0.04
Blood Eosinophils	$N*10^3$	0.04	0.01	0.04	0.00	0.03	0.01
BAL Leptin	pg/ml	103.4	12.2	104.9	17.8	132.9	26.7
BAL Adiponectin	pg/ml	3158.9	481.0	2292.0	130.9	2380.2	317.6
BAL Fibrinogen	pg/ml	35.1	4.5	50.9	4.6	47.4	5.0
BAL PAI-1	pg/ml	210.0	16.3	256.9	28.4	550.4	110.0
BAL IL-1α	pg/ml	0.2	0.0	0.2	0.0	0.2	0.0
BAL IL-1β	pg/ml	0.4	0.1	1.1	0.2	3.0	1.1
BAL IL-4	pg/ml	0.7	0.0	0.7	0.0	0.7	0.0
BAL IL-5	pg/ml	0.2	0.0	4.2	1.0	5.4	1.1
BAL IL-6	pg/ml	0.1	0.0	1.8	0.9	10.4	2.6
BAL IL-10	pg/ml	0.3	0.0	0.4	0.1	0.3	0.0
BAL Il-12p40	pg/ml	12.3	2.8	7.7	1.4	14.5	1.6
BAL IL-12p70	pg/ml	2.0	0.1	2.7	0.7	1.9	0.1
BAL IFNγ	pg/ml	0.4	0.1	0.4	0.1	0.1	0.0
BAL TNFα	pg/ml	1.6	0.1	1.6	0.1	1.6	0.1
BAL G-CSF	pg/ml	0.7	0.1	6.7	2.6	85.2	26.7
BAL CXCL1	pg/ml	1.4	0.1	3.0	0.6	43.0	7.1
BAL CCL5	pg/ml	0.0	0.0	0.0	0.0	0.0	0.0
BAL-MIP-2	pg/ml	0.5	0.0	2.4	0.3	16.5	2.3
Serum Leptin	pg/ml	718.9	111.1	1105.5	228.2	1762.7	366.4
SerumAdiponectin	pg/ml	3508.9	511.9	3110.7	347.8	3946.7	413.0
Serum Fibrinogen	pg/ml	84.7	21.2	71.6	28.5	201.1	54.0
Serum PAI-1	pg/ml	718.9	111.1	1105.5	228.2	1762.7	366.4
Serum IL-1α	pg/ml	32.9	4.1	34.7	5.9	31.3	7.4
Serum IL-1β	pg/ml	440.2	77.2	369.6	47.2	390.9	28.9
Serum IL-4	pg/ml	2.4	0.1	12.3	8.7	3.3	0.7
Serum IL-5	pg/ml	10.1	1.3	21.5	4.2	39.1	12.2
Serum IL-6	pg/ml	9.0	2.4	18.9	9.0	12.5	2.1

Serum IL-10	pg/ml	30.8	4.2	45.5	6.1	46.0	6.0
Serum Il-12p40	pg/ml	412.4	64.1	277.1	53.8	325.6	82.8
Serum IL-12p70	pg/ml	50.4	8.5	67.2	9.0	68.3	11.4
Serum IFNγ	pg/ml	50.0	28.3	15.1	2.2	12.4	2.0
Serum TNFα	pg/ml	171.5	60.0	126.6	25.9	138.9	15.7
Serum G-CSF	pg/ml	84.3	9.4	202.9	51.1	586.0	356.6
Serum CXCL1	pg/ml	19.7	7.6	49.4	21.8	132.3	77.7
Serum CCL5	pg/ml	49.4	5.6	47.0	4.5	41.0	4.0
Serum-MIP-2	pg/ml	18.7	0.9	16.8	1.3	30.5	12.3
		**Cafeteria**					
**Variable investigated**	**Unit**	**HCC Value**	**SEM**	**SHAM Value**	**SEM**	**CNP Value**	**SEM**
Body Mass Gain	g	7.4	0.6	8.0	0.6	7.6	0.7
Fat Mass Gain	g	4.1	0.2	4.8	0.4	4.4	0.3
Lean Mass Gain	g	3.2	0.5	3.1	0.5	3.1	0.4
BAL Volume	ml	10.2	0.3	10.9	0.4	11.0	0.2
BAL Cells	$N*10^6$	0.5	0.0	0.7	0.1	0.8	0.1
BAL Macrophages	$N*10^6$	0.5	0.0	0.6	0.1	0.6	0.1
BAL Lymphocytes	$N*10^6$	0.012	0.002	0.010	0.002	0.014	0.003
BAL Neutrophils	$N*10^6$	0.0014	0.0004	0.0415	0.0160	0.1771	0.0229
BAL Eosinophils	$N*10^6$	0.0005	0.0003	0.0033	0.0024	0.0032	0.0013
BAL Protein	µg/ml	113.7	10.4	122.3	10.4	149.0	10.1
BAL LDH	U/ml	0.15	0.03	0.10	0.02	0.14	0.02
White Blood Cells	$N*10^3$	1.8	0.2	1.9	0.2	2.7	0.3
Blood Neutrophils	$N*10^3$	0.32	0.08	0.48	0.08	0.75	0.19
Blood Lymphocytes	$N*10^3$	1.45	0.10	1.31	0.15	1.82	0.20
Blood Monocytes	$N*10^3$	0.04	0.01	0.09	0.02	0.07	0.01
Blood Eosinophils	$N*10^3$	0.02	0.00	0.03	0.01	0.03	0.00
BAL Leptin	pg/ml	188.5	35.9	135.3	18.7	175.6	16.8
BAL Adiponectin	pg/ml	3075.3	723.0	3514.4	573.8	3625.1	328.3
BAL Fibrinogen	pg/ml	32.1	3.8	59.0	7.4	69.0	11.0
BAL PAI-1	pg/ml	202.7	27.1	380.1	106.8	850.4	235.3
BAL IL-1α	pg/ml	0.2	0.0	0.2	0.0	0.2	0.0
BAL IL-1β	pg/ml	0.6	0.2	1.7	0.5	2.2	0.3
BAL IL-4	pg/ml	0.7	0.0	0.7	0.0	0.9	0.2
BAL IL-5	pg/ml	0.2	0.0	4.1	2.9	7.6	1.7
BAL IL-6	pg/ml	0.1	0.0	1.7	0.8	9.1	2.6
BAL IL-10	pg/ml	0.3	0.0	0.3	0.0	0.3	0.0
BAL Il-12p40	pg/ml	8.7	0.9	5.9	0.7	18.4	3.5
BAL IL-12p70	pg/ml	2.0	0.1	1.8	0.1	1.9	0.1
BAL IFNγ	pg/ml	0.1	0.0	0.2	0.0	0.2	0.0
BAL TNFα	pg/ml	1.6	0.1	1.6	0.1	1.6	0.1
BAL G-CSF	pg/ml	0.6	0.0	13.0	5.6	79.8	7.9
BAL CXCL1	pg/ml	1.5	0.1	4.2	1.2	42.7	7.5
BAL CCL5	pg/ml	0.0	0.0	0.0	0.0	0.0	0.0
BAL-MIP-2	pg/ml	0.5	0.1	4.3	1.3	12.7	1.0
Serum Leptin	pg/ml	780.2	154.6	1059.4	176.8	1148.3	275.1
SerumAdiponectin	pg/ml	3817.8	381.2	3750.5	610.0	3768.3	673.8
Serum Fibrinogen	pg/ml	92.2	22.4	103.3	52.2	163.4	63.4
Serum PAI-1	pg/ml	780.2	154.6	1059.4	176.8	1148.3	275.1
Serum IL-1α	pg/ml	26.3	5.5	29.1	7.5	32.5	7.6
Serum IL-1β	pg/ml	286.7	23.4	270.4	24.9	390.6	54.7
Serum IL-4	pg/ml	2.3	0.0	2.3	0.0	3.5	0.9
Serum IL-5	pg/ml	8.6	1.2	13.8	2.0	33.0	8.5
Serum IL-6	pg/ml	7.0	1.1	15.4	3.0	16.7	6.4

Serum IL-10	pg/ml	35.2	3.4	31.2	3.4	48.6	8.6
Serum Il-12p40	pg/ml	394.8	53.2	213.2	60.4	394.8	131.4
Serum IL-12p70	pg/ml	55.0	5.9	35.0	5.4	63.3	12.0
Serum IFNγ	pg/ml	16.6	2.8	10.4	2.3	18.6	3.9
Serum TNFα	pg/ml	114.0	16.1	69.4	10.8	139.7	25.0
Serum G-CSF	pg/ml	153.2	20.5	343.4	76.9	423.2	80.6
Serum CXCL1	pg/ml	35.1	16.5	55.4	9.5	64.3	25.9
Serum CCL5	pg/ml	41.2	3.9	41.3	3.8	42.5	3.7
Serum-MIP-2	pg/ml	17.3	2.6	13.1	1.7	15.4	2.1

		**High Fat**					
**Variable investigated**	**Unit**	**HCC Value**	**SEM**	**SHAM Value**	**SEM**	**CNP Value**	**SEM**
Body Mass Gain	g	6.5	0.7	5.5	0.4	6.8	0.5
Fat Mass Gain	g	4.3	0.5	3.7	0.2	3.8	0.2
Lean Mass Gain	g	2.4	0.3	1.8	0.3	3.1	0.5
BAL Volume	ml	10.9	0.5	10.9	0.3	10.8	0.5
BAL Cells	$N*10^6$	0.6	0.1	0.6	0.0	0.7	0.1
BAL Macrophages	$N*10^6$	0.6	0.1	0.5	0.0	0.5	0.1
BAL Lymphocytes	$N*10^6$	0.015	0.002	0.007	0.001	0.014	0.002
BAL Neutrophils	$N*10^6$	0.0009	0.0004	0.0419	0.0136	0.1587	0.0257
BAL Eosinophils	$N*10^6$	0.0006	0.0003	0.0014	0.0006	0.0103	0.0056
BAL Protein	µg/ml	123.7	14.8	131.9	16.1	152.2	17.1
BAL LDH	U/ml	0.13	0.01	0.12	0.02	0.14	0.02
White Blood Cells	$N*10^3$	2.6	0.1	3.9	0.6	4.3	0.4
Blood Neutrophils	$N*10^3$	0.35	0.05	0.88	0.20	0.80	0.14
Blood Lymphocytes	$N*10^3$	2.13	0.10	2.85	0.48	3.39	0.38
Blood Monocytes	$N*10^3$	0.04	0.01	0.13	0.05	0.09	0.02
Blood Eosinophils	$N*10^3$	0.03	0.01	0.04	0.01	0.04	0.01
BAL Leptin	pg/ml	163.1	14.5	190.6	38.2	139.6	16.1
BAL Adiponectin	pg/ml	2807.2	259.2	2967.3	567.9	3670.8	637.1
BAL Fibrinogen	pg/ml	37.9	4.9	35.3	6.8	50.4	8.4
BAL PAI-1	pg/ml	187.0	22.6	508.4	175.1	685.9	172.4
BAL IL-1α	pg/ml	0.2	0.0	0.2	0.0	0.2	0.0
BAL IL-1β	pg/ml	0.5	0.1	1.1	0.4	1.4	0.2
BAL IL-4	pg/ml	0.7	0.0	0.7	0.0	0.9	0.1
BAL IL-5	pg/ml	0.2	0.0	2.8	1.2	5.7	2.0
BAL IL-6	pg/ml	0.1	0.0	9.8	8.8	6.0	1.0
BAL IL-10	pg/ml	0.3	0.0	0.3	0.0	0.3	0.0
BAL Il-12p40	pg/ml	10.8	1.1	6.2	1.6	18.1	2.4
BAL IL-12p70	pg/ml	2.0	0.1	1.9	0.1	1.9	0.1
BAL IFNγ	pg/ml	0.1	0.0	0.2	0.0	0.1	0.0
BAL TNFα	pg/ml	1.6	0.1	5.2	3.4	1.6	0.1
BAL G-CSF	pg/ml	0.8	0.1	19.6	15.6	46.2	7.7
BAL CXCL1	pg/ml	3.8	1.1	4.0	1.9	29.6	7.2
BAL CCL5	pg/ml	0.0	0.0	0.1	0.0	0.0	0.0
BAL-MIP-2	pg/ml	1.6	0.5	4.3	2.0	9.8	1.4
Serum Leptin	pg/ml	1104.2	177.4	2189.1	758.2	909.7	158.5
SerumAdiponectin	pg/ml	3401.0	257.5	2593.1	327.7	2583.9	377.1
Serum Fibrinogen	pg/ml	63.1	27.6	30.8	15.5	96.2	27.9
Serum PAI-1	pg/ml	1104.2	177.4	2189.1	758.2	909.7	158.5
Serum IL-1α	pg/ml	31.3	6.8	52.6	8.9	49.7	15.0
Serum IL-1β	pg/ml	354.4	61.1	276.7	38.1	493.5	112.9
Serum IL-4	pg/ml	2.9	0.3	2.5	0.1	2.6	0.1
Serum IL-5	pg/ml	11.5	2.6	37.1	11.2	51.6	23.5
Serum IL-6	pg/ml	8.9	2.6	46.6	27.3	13.6	2.1

Serum IL-10	pg/ml	48.4	5.7	41.9	9.1	52.8	6.3
Serum Il-12p40	pg/ml	427.9	62.3	433.5	100.1	380.8	49.5
Serum IL-12p70	pg/ml	58.7	13.4	54.1	10.5	69.0	9.5
Serum IFNγ	pg/ml	50.6	27.0	10.6	1.9	17.2	2.6
Serum TNFα	pg/ml	136.2	30.4	134.5	38.1	152.9	40.1
Serum G-CSF	pg/ml	269.4	51.7	342.5	80.5	312.8	65.5
Serum CXCL1	pg/ml	90.1	23.8	115.6	48.2	72.7	22.1
Serum CCL5	pg/ml	72.6	11.4	63.3	12.6	55.4	6.0
Serum-MIP-2	pg/ml	33.7	4.4	17.1	3.0	19.1	2.3
